# Beyond the norm: exploring the uncommon squamous cell carcinoma of the prostate using a Saudi tumor registry

**DOI:** 10.1097/MS9.0000000000001220

**Published:** 2023-10-02

**Authors:** Ahmed Alasker, Areez Shafqat, Belal Nedal Sabbah, Mohammad Alghafees, Aljoharah Adeeb Al Saud, Nasouh Aljabi, Khalid Mazin Altaweel, Raghad Alhumaidan, Ziyad F. Musalli, Abdulaziz Almanie, Rakan Abu Alqam, Salman Bin Ofisan

**Affiliations:** a College of Medicine, King Saud bin Abdulaziz University for Health Sciences; b King Abdulaziz Medical City; c King Abdullah International Medical Research Center; d College of Medicine, Alfaisal University; e Security Forces Hospital, Riyadh; f College of Medicine, King Abdulaziz University, Jeddah; g College of Medicine, Prince Sattam Bin Abdulaziz University, Al-Kharj, Saudi Arabia

## Abstract

**Background:**

Squamous cell carcinoma (SCC) of the prostate has limited treatment choices and portends a dismal prognosis with an average survival time of ~14-months. This study provides a descriptive overview of SCC of the prostate in Saudi Arabia.

**Materials and Methods:**

A retrospective cohort study of patients diagnosed with prostatic SCC between 1 January 2008 and 31 December 2017. Information on demographic and tumor characteristics and the survival of patients was collected from the Saudi Cancer Registry. Survival was depicted through Kaplan–Meier plots. Fisher’s exact test was used to assess the association between categorical variables and death, while a Wilcoxon rank sum test was applied for numerical variables.

**Results:**

Out of a larger subset of 3607 patients, 16 patients were diagnosed with prostatic SCC, of which half resided in the Central region (50.0%) and most (81.2%) were aged greater than or equal to 60 years. Most patients (62.6%) had poorly differentiated (grade III, 43.8%) lesions, and 50% of cases were metastatic at diagnosis. 62.5% of patients died, all residing in the Eastern and Central regions. Regional extension (75.0%) and distant metastasis (87.5%) were significantly associated with death compared to localized lesions (0.0%) (*P*=0.022). The 5-year survival rate in our study was 33%.

**Conclusion:**

The present study is the first to describe the characteristics of prostatic SCC in Saudi Arabia. Our results are consistent with prior studies showing that prostatic SCC is often high-grade and metastatic at diagnosis, conferring a poor prognosis.

Highlights

- This study provides a comprehensive analysis of 16 cases of squamous cell carcinoma (SCC) of the prostate, an uncommon and aggressive variant of prostate cancer, using data from the Saudi Cancer Registry.
- The study reveals that SCC predominantly affects males above the age of 60, with high-grade lesions and frequent distant metastasis. Histological examination remains the primary mode of diagnosis.
- Despite the challenging prognosis, the 5-year survival rate for SCC patients in this study was 33%, which is higher than previously reported. Factors such as place of residence and tumor extent were found to be significant predictors of mortality.

## Background

Prostate cancer stands as the most prevalent cancer in males and constitutes the second-leading cause of cancer-related mortality among men^1^. The majority of prostate cancer cases are adenocarcinomas, whereas a rare and aggressive variant, squamous cell carcinoma (SCC), accounts for 05–1% of prostate cancer cases^2,3^. The literature on primary SCC of the prostate is limited, with fewer than 100 reported cases^4^.

The exact cause of SCC of the prostate remains unknown, with the prostatic urethral urothelium, periurethral ductal epithelium, prostatic acinar epithelium, or radiation- or endocrine-therapy-induced differentiation of adenocarcinoma cells all being implicated as potential origins of the cancer^4–8^. Patients with prostatic SCC often present with obstructive symptoms, such as weak stream, straining, and hesitancy, as well as metastatic symptoms like bone pain from osteolytic metastases, unlike the osteoblastic metastases that are typically a feature of adenocarcinomas^9,10^.

Histological examination of a biopsy or surgical specimen remains the mainstay of diagnosis^11^. Mott proposed the following criteria to identify primary SCC of the prostate: (i) the presence of clearly malignant invasive neoplasm with disordered growth and cellular anaplasia; (ii) definite squamous features of keratinization, squamous pearls and/or numerous distinct intercellular bridges; (iii) the absence of glandular or acinar pattern (indicative of adenocarcinoma with squamous metaplasia); (iv) no prior estrogen therapy; and (v) the absence of primary squamous cancer elsewhere, particularly in the bladder^12^. These criteria help distinguish prostatic SCC from non-neoplastic squamous metaplasia possibly secondary to an infarction, acute/chronic prostatitis, and granulomatous prostatitis due to Bacillus Calmette–Guérin (BCG), estrogen, or radiation therapy^12^.

Due to its aggressive nature and resistance to standard therapies, SCC of the prostate has limited treatment choices and portends a dismal prognosis with an average survival time of ~14-months^13^. Radiation therapy, chemotherapy, and radical prostatectomy have all been tried with limited success^10,14,15^.

This study provides a descriptive overview of SCC of the prostate with a comprehensive analysis of 16 cases retrieved from the Saudi Cancer Registry (SCR) in Saudi Arabia.

## Methods

This retrospective cohort study included all patients diagnosed with prostatic SCC between 1 January 2008 and 31 December 2017. Patients with secondary lesions metastasizing to the prostate were excluded. The data was collected from the SCR, a registry that collects tumor data from all private, military, and health ministry hospitals across five regional offices in Saudi Arabia. The data analysis and periodic reporting take place at the main office in Riyadh. This work has been reported in line with the STROCSS criteria^16^. This study has been registered into the research registry [researchregistry9189].

### Statistical analysis

All statistical analyses was performed using RStudio (R version 4.2.2.). Categorical variables were expressed as frequencies and percentages, while numerical variables were presented as the median and interquartile range (IQR). Survival analysis was conducted using the Kaplan–Meier method. Due to the large number of variables with zero frequencies and empty rows that could limit the validity of a Cox regression analysis, we opted to employ an inferential test to assess factors associated with cancer-related mortality. Fisher’s exact test was used to assess the association between categorical variables and death, while a Wilcoxon rank sum test was applied for numerical variables. Statistical significance was defined as *P* <0.05.

## Results

### Demographic characteristics

**Figure 1:**
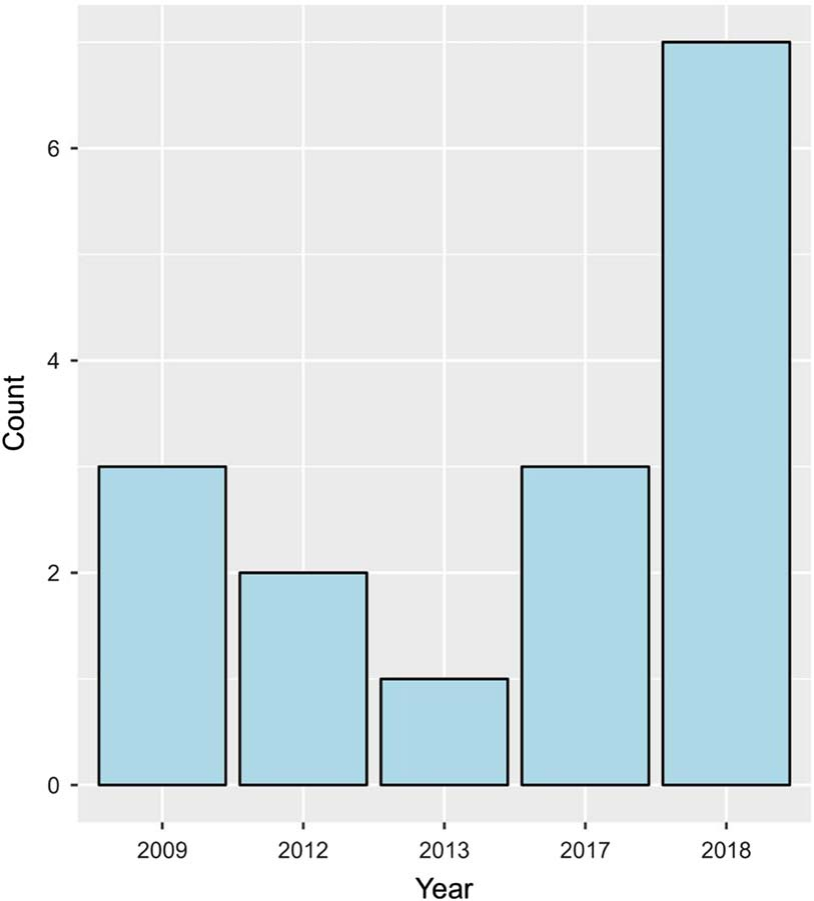
The frequencies of patients with squamous cell carcinoma who had been diagnosed at different time points.

**Figure 2:**
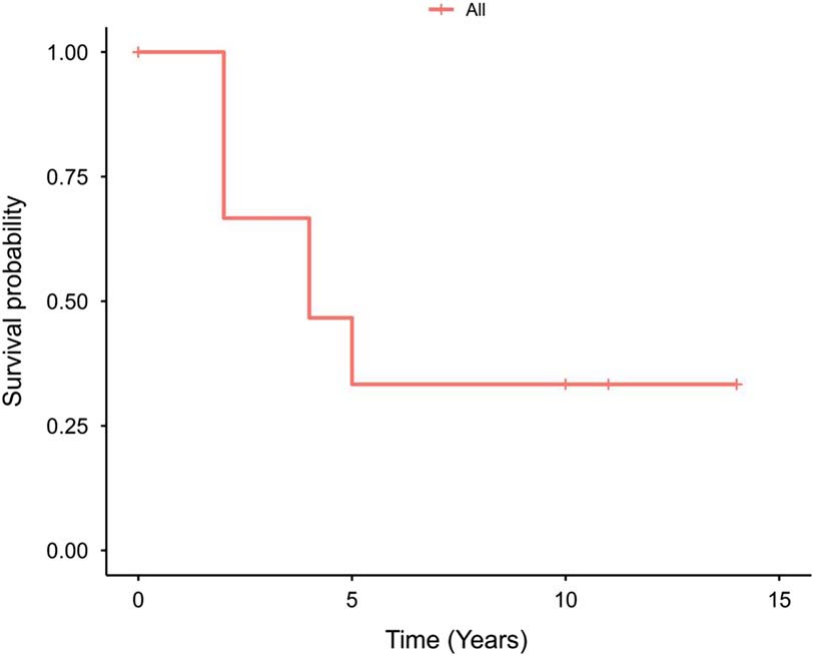
A Kaplan–Meier curve for the overall survival of patients with squamous cell carcinoma.

In this study, we focused on a subset of a larger dataset of 3607 patients with four types of cancers: acinar adenocarcinoma (*n*=3374, 93.5%), ductal adenocarcinoma (*n*=209, 5.8%), embryonal rhabdomyosarcoma (*n*=8, 0.2%) and SCC (*n*=16, 0.4%). Our analysis specifically examined 16 patients with SCC of the prostate and compared their survival rates to those with other types of cancer. Among patients with SCC, half of them resided in the Central region (50.0%), and most (81.2%) were aged greater than or equal to 60 years. Detailed demographic characteristics are presented in Table 1. The majority of our patients (*n*=7, 43.8%) were diagnosed with the disease in 2018 (Fig. 1). The median (IQR) interval between diagnosis and last contact or death was 4.0 years (2.0–10.2) (Fig. 2).

**Table 1:** Demographic characteristics

Parameter	Category	*N* (%)
Place of residency	Central region	8 (50.0)
	Eastern region	4 (25.0)
	Western region	3 (18.8)
	Northern region	0 (0.0)
	Southern region	1 (6.2)
Age group	≤18 Years	0 (0.0)
	19–39 years	0 (0.0)
	40–59 years	3 (18.8)
	≥60 years	13 (81.2)
Marital status	Single	0 (0.0)
	Married	16 (100.0)
	Divorced	0 (0.0)
	Widowed	0 (0.0)

### Tumor characteristics

Only one patient (6.2%) had a grade I tumor, whereas grades II (five patients, 31.2%), III (seven patients, 43.8%), and IV (three patients, 18.8%) tumors were more prevalent. Localized tumors were detected in 25.0% of patients, whereas distant metastasis was reported among 50.0% of cases. The diagnosis was based on histological testing of the primary lesion in most patients (87.5%) and the histology of metastases in 12.5% of cases. Further details on tumor characteristics can be found in Table 2.

**Table 2:** Characteristics of the tumors

Parameter	Category	*N* (%)
Grade	Grade I (well diff)	1 (6.2)
	Grade II (mod diff)	5 (31.2)
	Grade III (poor diff)	7 (43.8)
	Grade IV (undiff anaplastic)	3 (18.8)
Extension	Localized	4 (25.0)
	Regional: lymph node	0 (0.0)
	Regional: direct extension	4 (25.0)
	Distant metastasis	8 (50.0)
Base of diagnosis	Laboratory test	0 (0.0)
	Histology of primary	14 (87.5)
	Histology of metastases	2 (12.5)
	Autopsy	0 (0.0)
Status	Alive	6 (37.5)
	Dead	10 (62.5)
Interval between diagnosis and event	Median (IQR)	4.0 (2.0, 10.2)

### Analysis of mortality and associated factors

Among patients with SCC of the prostate, death occurred in 10 patients (62.5%). Residing in the Eastern and Central regions was significantly associated with death (100.0 and 75.0%, respectively) compared to other provinces (0% in all) (*P*=0.018). This phenomenon could be attributed to the fact that the most challenging cases are generally referred to these regions owing to the presence of the largest hospitals. Additionally, the waiting lists are very long in these hospitals. Regional extension (75.0%) and distant metastasis (87.5%) were also significantly associated with death compared to localized lesions (0.0%) (*P*=0.022). The time interval between diagnosis and last contact or death was significantly shorter among dead patients (median = 3.0 years, IQR = 2.0 to 4.0) compared to alive patients (median = 12.5 years, IQR = 10.2–14.0, *P* = 0.031, Table 3).

**Table 3:** Factors associated with death among patients with squamous cell carcinoma

Parameter	Category	Alive, *N*=6	Dead, *N*=10	*P*
Place of residency	Central region	2 (25.0%)	6 (75.0%)	0.018
	Eastern region	0 (0.0%)	4 (100.0%)	
	Western region	3 (100.0%)	0 (0.0%)	
	Northern region	0 (NA%)	0 (NA%)	
	Southern region	1 (100.0%)	0 (0.0%)	
Age group	40–59 years	0 (0.0%)	3 (100.0%)	0.250
	≥60 years	6 (46.2%)	7 (53.8%)	
Grade	Grade I	1 (100.0%)	0 (0.0%)	0.266
	Grade II	3 (60.0%)	2 (40.0%)	
	Grade III	2 (28.6%)	5 (71.4%)	
	Grade IV	0 (0.0%)	3 (100.0%)	
Extension	Localized	4 (100.0%)	0 (0.0%)	0.022
	Regional: direct extension	1 (25.0%)	3 (75.0%)	
	Distant metastasis	1 (12.5%)	7 (87.5%)	
Base of diagnosis	Histology of primary	4 (28.6%)	10 (71.4%)	0.125
	Histology of metastases	2 (100.0%)	0 (0.0%)	
Interval between diagnosis and event	Median (IQR)	12.5 (10.2, 14.0)	3.0 (2.0, 4.0)	0.031

### Survival analysis

**Figure 3:**
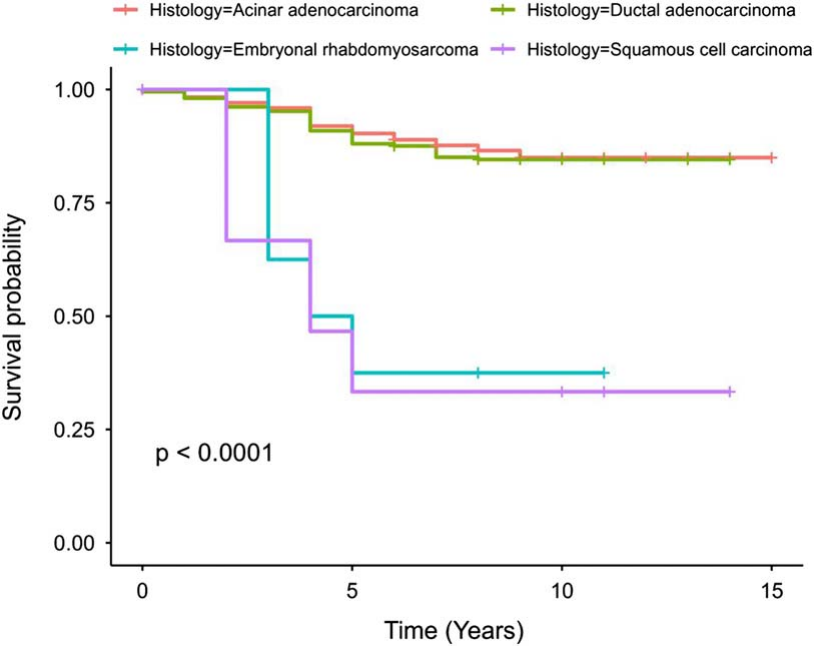
A Kaplan–Meier curve for the survival of patients with four types of cancer, including acinar adenocarcinoma, ductal adenocarcinoma, embryonal rhabdomyosarcoma and squamous cell carcinoma.

The survival of patients with SCC was compared to three histopathological types included in our dataset. The 5-year survival rate was 90.3% (95% CI: 89.3–91.3) for acinar adenocarcinoma, 88.0% (95% CI: 83.7–92.5) for ductal adenocarcinoma, 37.5% (95% CI: 15.3–91.7) for embryonal rhabdomyosarcoma and 33.3% (95% CI: 16.3–68.2) for SCC. The difference in survival rates among these different histopathological types was statistically significant (*P* <0.0001, Fig. 3).

## Discussion

Our study provides a descriptive overview and survival analysis of 16 cases of SCC of the prostate from the SCR in Saudi Arabia. Consistent with prior studies, our studies indicate that SCC predominantly affects males above 60 years old^17^. Most of our patients presented with high-grade lesions (≥grade III) and distant metastasis were common (50%) compared to localized tumors (25% of patients), which is also in agreement with prior studies where this type of tumor was found to be high-grade at presentation and metastasize most frequently to regional lymph nodes and bone, with the lungs, liver, and brain being less common sites^17,18^. Histological examination was the primary mode of diagnosing the primary lesions (87.5%), similar to prior case studies^4,19,20^.

Our study revealed a high mortality rate in SCC patients (62.5%), with place of residence and tumor extent being the strongest predictors of mortality. Encouragingly, the 5-year survival rate for the individuals in our study was 33%, which is significantly higher than most studies as per a meta-analysis that showed the 1-year survival of prostatic SCC to be 32% and a 3-year survival of only 9%^17^. Treatment options for SCC of the prostate are limited, with the contemporary literature reporting a mix of chemotherapy, radiotherapy, and prostatectomy use, either in combinations or alone, all with limited success^17,21^. However, long-term survival has recently been reported in recent independent case reports with a combination of radiotherapy to the whole pelvis and prostate along with a chemotherapy regimen of docetaxel, cisplatin, and 5-fluorouracil^4,22,23^.

There are several limitations to our study, most notably the small sample size. A more comprehensive retrospective study with a larger sample size would provide a better understanding of the demographic and clinical characteristics, mortality rates, management, and prognosis of prostatic SCC among Saudi citizens. Furthermore, issues such as underreporting, lack of data on clinical characteristics, incomplete data representation in the TNM classification, distant metastasis, and failure to include crucial factors like specific treatment strategy are limitations in our study that should be addressed by future studies. Lastly, we could not compare differences in demographic and clinical characteristics between different geographical regions to the further our findings of region-specific mortality.

## Conclusion

Our findings are in agreement with prior research showing that prostatic SCC is associated with a high-grade and metastasis at diagnosis. Place of residence, particularly in the Eastern and Central regions of Saudi Arabia, was a significant predictor of mortality. Further research is needed to identify the optimal treatment regimen of these patients.

## Ethics approval

Ethical approval (NRC21R/499/11) was obtained from King Abdullah International Medical Research Center (KAIMRC) prior to the beginning of this study. This study is a secondary analysis.

## Consent to participate

Written informed consent was obtained from the patient for publication and any accompanying images. A copy of the written consent is available for review by the Editor-in-Chief of this journal on request.

## Sources of funding

This research did not receive any funding from institutions in the public, commercial, or not-for-profit sectors.

## Author contribution

All authors contributed to the research and/or preparation of the manuscript. A.A., M.A., A.S., and B.N.S.: participated in the study design and wrote the first draft of the manuscript; A.A., A.S., B.N.S., M.A., A.A.A.S., N.A., K.M.A., R.A., Z.F.M., A.B.A., R.A.A., and S.B.O.: collected and processed the data; M.A.: participated in the study design and performed the statistical analyses; M.A., A.S., and B.N.S.: reviewed and finalized the manuscript. All authors read and approved the final manuscript.

## Conflicts of interest disclosures

The authors declare that there are no conflicts of interest.

## Research registration unique identifying number (UIN)

researchregistry9189.

## Guarantor

Mohammad Alghafees.

## Availability of data and material

Not applicable.

## Provenance and peer review

Not commissioned, externally peer reviewed.
